# Magneto-Mechanical and Thermal Properties of Nd-Fe-B-Epoxy-Bonded Composite Materials

**DOI:** 10.3390/polym15081894

**Published:** 2023-04-14

**Authors:** Aleksandar Grujić, Dragutin Nedeljković, Jasna Stajić-Trošić, Mirko Z. Stijepović, Sabla Alnouri, Srdjan Perišić

**Affiliations:** 1 Institute of Chemistry, Technology and Metallurgy, National Institute of the Republic of Serbia, University of Belgrade, Njegoševa 12, 11000 Belgrade, Serbia; dragutin@tmf.bg.ac.rs (D.N.); jtrosic@tmf.bg.ac.rs (J.S.-T.); 2 Faculty of Technology and Metallurgy, University of Belgrade, 11000 Belgrade, Serbia; 3 Gas Processing Centre, College of Engineering, Qatar University, Doha P.O. Box 2713, Qatar; 4 Innovation Center of Faculty of Technology and Metallurgy, University of Belgrade, 11000 Belgrade, Serbia

**Keywords:** Nd-Fe-B, epoxy, composites, bonded magnets, magnetic materials, DMA, impact toughness

## Abstract

Polymer-bonded magnets are a class of composite material that combines the magnetic properties of metal particles and the molding possibility of a polymeric matrix. This class of materials has shown huge potential for various applications in industry and engineering. Traditional research in this field has so far mainly focused on mechanical, electrical or magnetic properties of the composite, or on particle size and distribution. This examination of synthesized Nd-Fe-B-epoxy composite materials includes the mutual comparison of impact toughness, fatigue, and the structural, thermal, dynamic-mechanical, and magnetic behavior of materials with different content of magnetic Nd-Fe-B particles, in a wide range from 5 to 95 wt.%. This paper tests the influence of the Nd-Fe-B content on impacting the toughness of the composite material, as this relationship has not been tested before. The results show that impact toughness decreases, while magnetic properties increase, along with increasing content of Nd-Fe-B. Based on the observed trends, selected samples have been analyzed in terms of crack growth rate behavior. Analysis of the fracture surface morphology reveals the formation of a stable and homogeneous composite material. The synthesis route, the applied methods of characterization and analysis, and the comparison of the obtained results can provide a composite material with optimum properties for a specific purpose.

## 1. Introduction

Polymer Bonded Magnets (or PBMs) based on Nd-Fe-B alloys are high-performance magnetic materials with high dimensional accuracy [1,2]. They are corrosion resistant materials that possess excellent magnetic properties [1,3]. They can be magnetized in axial, radial and planar directions. Produced PBMs have a smooth surface, a final shape which is achieved with no additional mechanical treatment, good homogeneity of magnetic particles, and high resistance to breaks or cracks. [4,5,6]. Moreover, since they can be easily molded into many different shapes and geometries, PBMs are very suitable for the manufacture of spindle drive motors [7] and hi-tech devices (such as cellular phones, computers, sensor systems, etc.) [2]. Broad PBM applications require processing of polymer composite materials with a wide range of magnetic filler content, under constant conditions [8].

Nd-Fe-B-epoxy-bonded composite is commonly produced by a compression molding process, due to the customized curing regime, high dimensional and high temperature stability, and the nature of the net chain structure of epoxy [5,6]. This production method includes thermosetting epoxy as a binding agent, and gives a bonded product with the highest magnetic properties, due to the possibility of operation with a high proportion of the magnetic medium [5,6]. The advantage of this type of PBM is the high density of magnetic particles, achieving good magnetic properties compared to PBMs with thermoplastic or rubber matrices [6]. However, compared to ferrite-bonded magnets, Nd-Fe-B-bonded magnets are considerably more expensive, have less stability (ferrites are oxides by themselves), and have a slightly lower operating temperature, but therefore superior magnetic properties [1]. Compared with sintered Nd-Fe-B magnets, PBMs have lower magnetic properties, because the density is lower due to the isolation of magnetic particles by a polymer [4]. Nevertheless, PBMs possess significant magnetic energy and a more affordable price, because they contain a smaller content of expensive rare earth than sintered magnets [5]. Research activities have also aimed at the development of bonded hybrid magnetic composite materials with improved dynamic-mechanical properties by substituting Nd-Fe-B with ferrite magnetic powders, or with improved magnetic properties, using powders based on neodymium and samarium [1,9,10].

Experimentally measured properties of the composite, such as maximum magnetic energy and impact toughness, are directly affected by magnetic particle content [4,11]. This is essential from an application standpoint, since a composite with an accurately determined composition could be applied for a specific purpose [6]. Moreover, results related to the crack growth rate in magnetic composites are not common in the literature, although this value is very important from an exploitation standpoint [12].

Most of the research studies which have been published so far in the field of PBMs have been directed towards assessing various PBM characteristics such as: filler particle size [13], tensile and flexural strength [14,15], corrosion resistance [3,16], dynamic and mechanical properties [17,18], magnetic alignment [19,20], and electrical and magnetic features [21,22]. It is worth mentioning that PBMs filled with Nd-Fe-B are suitable for environmentally friendly recycling at elevated temperatures, retaining good magnetic and mechanical performances, as reported by Gandha et al. [23]. However, very few studies assessing the impact toughness of magnetic materials have been found, with the exception of those involving commercial sintered magnets [24].

Generally speaking, the type of PBM processing technology to be used depends on many factors, such as the polymer type (e.g., thermosetting, thermoplastic, rubber, etc.), the polymer content, the filler properties and the filler content [1,25,26,27,28]. Moreover, different applications require different mechanical and magnetic properties in the final PBM product [18]. For instance, PBMs that are used for sensors and membranes often require a relatively small portion of magnetic materials, uniformly arranged in a polymer matrix [22,29]. In such systems with low magnetic filler fractions, the type of particle and particle size has a greater influence on the final properties of the composites than particle content [25]. In many other cases, achieving uniform particle distribution is also essential for attaining the desired mechanical and magnetic properties of the final PBM product. However, it is often very challenging to achieve uniform particle distribution during many processing situations. Particle size, particle distribution, and the ratio of the contents of filler particles to polymer matrix have a high impact on the filler-to-binder properties, the degree of particle alignment, the packing density, and the magnetic and dynamic mechanical properties, as previously reported [17].

It has been shown that PBMs with more than 98 wt.% of Nd-Fe-B often possess high magnetic and mechanical properties [15]. According to Zhang et al. [15], such properties can be induced using a compacting pressure that ranges from 350–700 MPa, and a compacting temperature that ranges from 90–200 °C. Composites with magnetic filler content ranging from 65–70 vol.% in epoxy resin show very good magnetic properties, especially when produced using compression molding techniques [30]. On the other hand, composites with lower magnetic filler content (often ranging from 40–60 vol.%) are better produced using injection techniques, which often utilize a thermoplastic polymer matrix [30]. Using mechanical stirring under sonication as synthesis method, Yunas et al. show that the magnetic property of PBMs is greatly associated with the amount of Nd-Fe-B particles embedded in the polymer matrix [31]. More precisely, they synthesized a magnetic composite actuator membrane capable of specific purposes, using composite with 6 wt.% Nd-Fe-B content. Different production techniques based on extrusion methods could be applied for rear-earth-epoxy composites with magnetic particle weight fraction ranging from 43 wt.% to 85 wt.% [28,32].

Various ratios of the Nd-Fe-B to polymer matrix have a direct impact on the structural, mechanical and magnetic properties of PBMs [1,9]. Sometimes, mechanical defects may appear in PBMs’ microstructure, which often leads to deformation or crack propagation. To reduce the possibility of damage, fatigue analysis can be used to give answers regarding PBMs’ behavior under variable loads. Fatigue mainly results in the failure of structures during their exploitation phase [33]. Linear elastic fracture mechanics (LEFM) could be applied for benchmarking fracture behavior and the initiation of cracks in epoxy composites [34,35]. LEFM assumes that stress intensity factor (K) uniquely defines the initial crack conditions, in addition to the crack growth conditions initiated by fatigue [34,36].

In alignment with previous research activities [14,17,37,38,39], this study demonstrates the effect of varying the Nd-Fe-B weight fraction on magnetic behavior, microstructure, thermal properties and impact toughness, as well as on the fatigue crack propagation of PBMs. A correlation between these properties has been developed and presented in this work for composites with various weight ratios of Nd-Fe-B to epoxy resin. The results obtained show the behavior of composites imposed by an external magnetic field, elevated temperatures, and different mechanical loads, and an analysis of their structural properties that varied with the content of magnetic Nd-Fe-B particles embedded in the polymer matrix, across a wide range from 5 to 95 wt.%.

## 2. Materials and Methods

### 2.1. Materials

**Figure 1:**
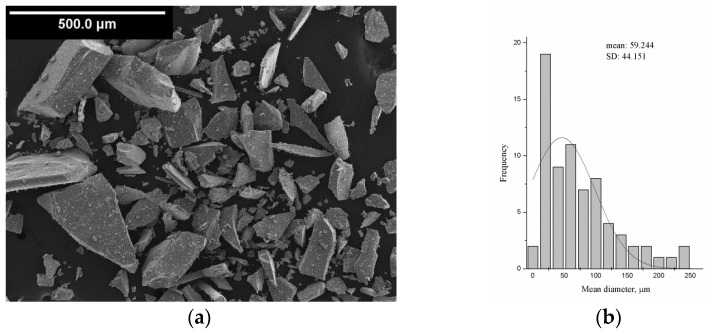
(**a**) SEM image, and (**b**) particle size histogram of used Nd-Fe-B alloy.

Stoichiometric Nd-Fe-B alloy was applied as magnetic filler for polymer composite materials production. The magnetic test, conducted on a 0.461 cm^2^, sample gives: (*BH*)*_max_* = 104.1 kJ/m^3^, *H_cb_* = 480 kA/m, *H_cj_* = 694 kA/m, *B_r_* = 0.818 T. Moreover, the chemical composition is as follows: Nd (21–25 wt.%), Co (5 wt.%), Zr (3–5 wt.%), B (1.5 wt.%) and Fe (balance) [38]. A SEM image of used particles and the respective particle size histogram are presented in Figure 1a,b.

A combination resin-hardener thermosetting epoxy binder has been used as the PBM matrix. The resin was obtained by mixing two types of bisphenol, A-type and F-type, and subsequently modified using a suitable dysfunctional reactive solvent, as reported elsewhere [39]. The hardener is a modified cycloaliphatic polyamine. The mixture’s pot life is 40 min at 20 °C. After 24 h, 80–90% solidification can be achieved. The minimum and maximum solidification temperatures are 10 °C and 40 °C, respectively, while the optimal solidification temperature ranges from 20–25 °C.

### 2.2. Preparation of Composites

**Figure 2:**
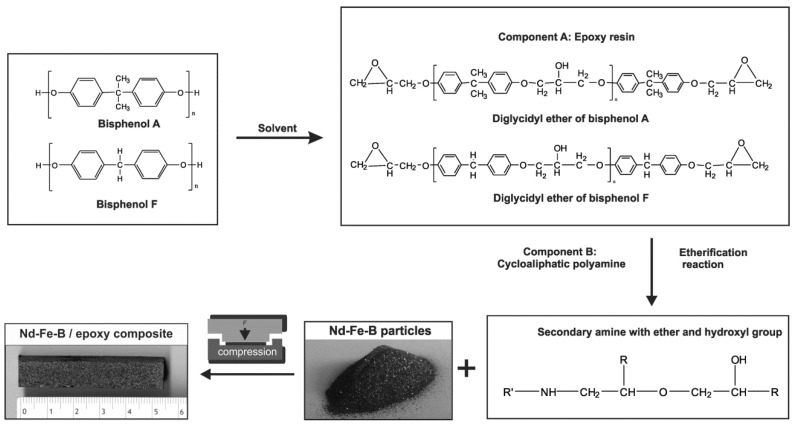
The mechanism of chemical reactions and production route.

PBMs of varying particle weight fraction (Nd-Fe-B) in the thermosetting matrix (epoxy) were synthesized by compression method (4 MPa) at ambient temperature. It should be noted that the pressure and operating temperature directly depends on determination of the rheological and thermal properties of the polymer [40]. The Nd-Fe-B filler contents varied from 5 to 95 wt.%. The Nd-Fe-B and epoxy resin were mixed using a mechanical stirrer for around 15 min under ambient temperature conditions. In order to prevent any magnetic particle oxidation, this process was conducted using an inert atmosphere. After a particular time in a vacuum chamber, the composite mixture was poured into the mold and compressed. After 5 h, the sample was taken out of the mold and left for 48 h to settle. The mechanism of the chemical reactions and production route is presented in Figure 2 [39]. During the compression process, all bubble inducing effects were minimized, and a uniform particle distribution was achieved.

### 2.3. Characterizing Methods

#### 2.3.1. Chemical Characterization

The Fourier-transform infrared (FT-IR) spectra of the samples in the KBr discs were recorded by a BOMEM spectrometer (from the Hartmann & Braun, MB series, 4 cm^−1^ resolution), using a transmission mode between 4000 and 400 cm^−1^.

#### 2.3.2. Thermal Analysis

Thermal analysis was conducted from room temperature to 600 °C at a heating rate of 10 °C/min under a nitrogen flow of 500 mL/min, using a Differential Scanning Calorimeter, DSC, Q100 TA Instruments.

The dynamic-mechanical properties and glass transition temperatures were examined using DMA Q800, TA Instruments, in a temperature range from room temperature to 100 °C at a heating rate of 3 °C/min. A three-point bend clamp with a 20 mm span width and rectangular-edge probe was used to test the prismatic samples of 36 × 12 × 3 mm at a frequency of 1 Hz.

#### 2.3.3. Surface Characterization

A Scanning Electron Microscope (SEM) JEOL JSM-5800-type was used for observing the morphology and the surface structure of the fractured samples imposed by impact testing. For enhanced conductivity, gold sputtering of samples was conducted by sputter coater POLARON SC 502-type. The time of sputtering was 15 s in two sections under 20 mA current. The thickness of the sputter coating was 5–10 nm.

#### 2.3.4. Impact Test

**Figure 3:**
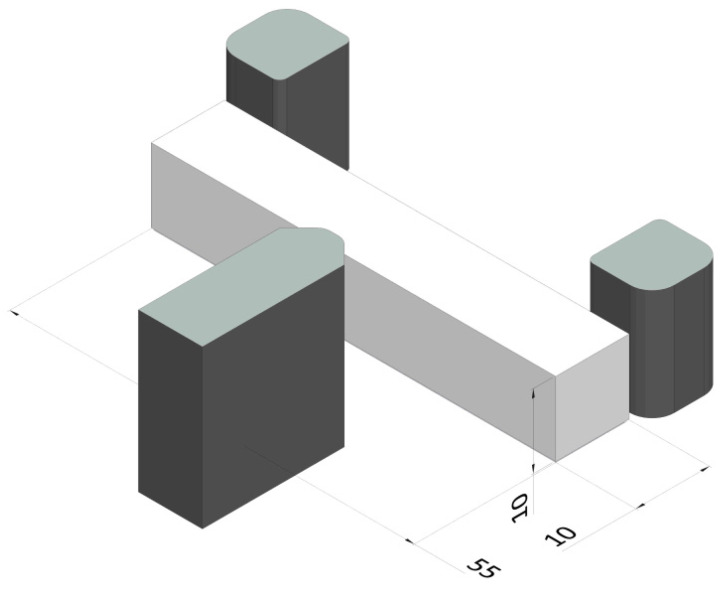
Schematic illustration of Charpy impact test with the geometry of the specimen.

Impact test was performed using a SCHENCK TREBEL 150J instrumented machine. Charpy composite specimens of 10 × 10 × 55 mm were tested at room temperature, according to the ASTM D E23-01 model illustrated in Figure 3 [41]. The support span length was 40 mm, the nose radius of the pendulum striker (hammer edge) was 8 mm, and the angle of fall was 45°.

#### 2.3.5. Crack Growth Rate Analysis

A high-frequency resonance pulsator (CRACKTRONIC) based on a three-point bending technique was used for monitoring the fatigue crack growth rate (da/dN). Sinusoidal cycles ranging from −70 to 70 Nm with a constant frequency of 135 Hz were applied. The calculation procedure for Δ*K* and da/dN was performed based on the ASTM E647-15e1 [42].

#### 2.3.6. Magnetic Measurements

Magnetic measurements were conducted using a SQUID magnetometer MPMS 5XL-type, with magnetic field strength between −5 and +5 T under room temperature conditions. The magnetic moment can be measured with great accuracy using this device. Hence, the demagnetization factor was neglected.

## 3. Results and Discussion

### 3.1. Chemical Properties

**Figure 4:**
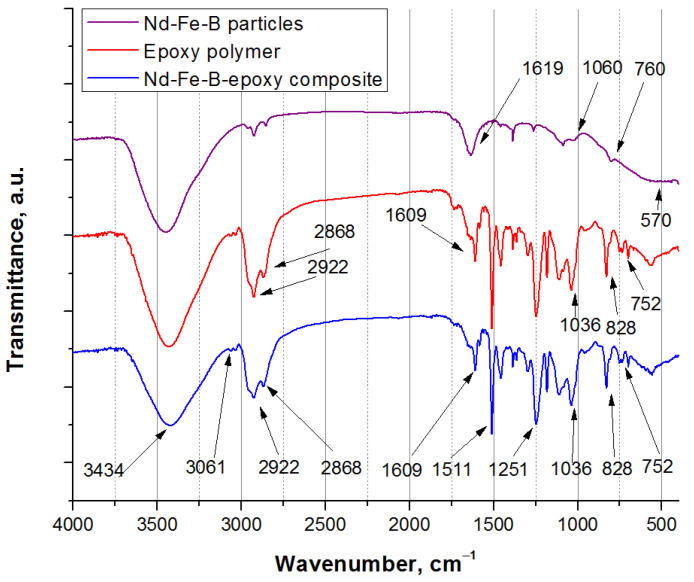
FT-IR spectra of composite material and components.

The obtained FT-IR spectra of the epoxy-bonded Nd-Fe-B composite material and the main components, epoxy resin and Nd-Fe-B alloy are presented in Figure 4. FT-IR spectra suggested the presence of non-covalent interactions between the magnetic fillers and the epoxy matrix. The presence of the broadband at 3434 cm^−1^ corresponds to O-H stretching vibrations of water and N-H stretching vibrations of the amine compound, while the characteristic band at 3061 cm^−1^ corresponds to C-H stretching vibrations of the epoxy ring [43]. The band at 2922 cm^−1^ corresponds to a C-H asymmetric stretching of CH_3_. Moreover, the characteristic band at 2868 cm^−1^ corresponds to C-H symmetric stretching of CH_2_. A C=C stretching of aromatic rings was also observed by the band at 1609 cm^−1^.

The band at 1511 cm^−1^ is a characteristic of aromatic C−C stretching. The two absorption bands at 1251 cm^−1^ and 828 cm^−1^ were associated with the most characteristic change, due to the C-O-C stretching vibrations of the epoxy ring [43]. Finally, the band at 1036 cm^−1^ corresponds to the stretching C-O-C of ethers, while the band observed at around 752 cm^−1^ is attributed to C-H bonding. The FT-IR spectrum of the Nd-Fe-B-epoxy composite was found to match that of the pure epoxy, i.e., no additional bands were detected.

The FT-IR spectrum of Nd-Fe-B shows bands for Fe vibrations at 570 cm^−1^ and B vibrations at 1060 cm^−1^ [29], while the O-H vibrations of moisture water are indicated at 3434 cm^−1^ and 1619 cm^−1^. In addition, the presence of some oxides probably induces the bands at around 760 cm^−1^ and 730 cm^−1^ [44].

### 3.2. Thermal Analysis Results

**Figure 5:**
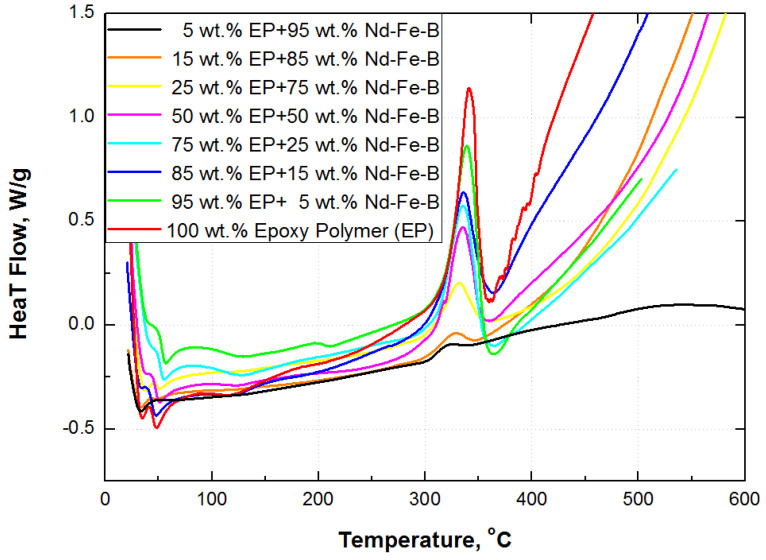
DSC curves of composites and pure epoxy resin.

Thermal behavior of polymer composites is essential from the operating point of view. Despite the high maximum energy product, applied magnetic particles have a low Curie temperature (around 312 °C) which limits their use at elevated temperatures [45]. On the other hand, the process of thermal degradation of epoxy resin is in a range from around 305 to 365 °C, as presented in Figure 5. The DSC curves for pure epoxy resin and composites with 5, 15, 25, 50, 75, 85 and 95 wt.% of magnetic filler are developed on the basis of previous research activities [9] and presented in Figure 5.

**Figure 6:**
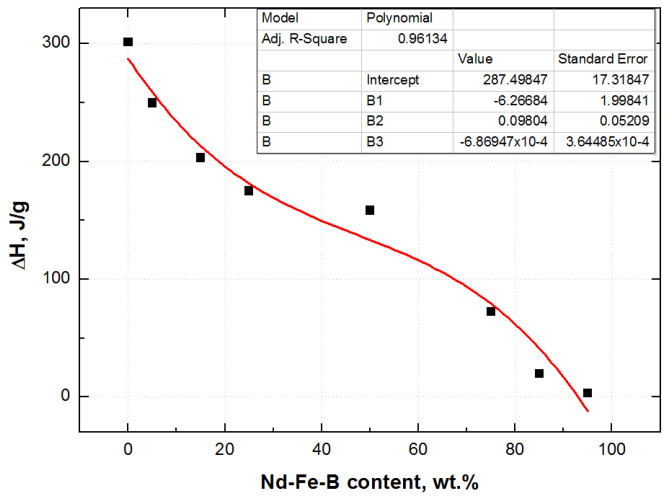
The function of the change of enthalpy with the weight fraction of Nd-Fe-B.

The temperature peak of the change of enthalpy shifts to the higher values for degradation temperature of composites with a lower weight fraction of magnetic filler, as presented in Figure 5. The pure epoxy resin and slightly filled composite have the highest change of enthalpy (identified as a red and green curve in Figure 5, respectively), while the highly filled composite, with 95 wt.% of Nd-Fe-B (black curve), has the lowest change of enthalpy (lowest peak). The change of enthalpy is calculated by integrating peak linearity in the region of thermal decomposition. A decreasing trend in the change of enthalpy with increasing wt.% of Nd-Fe-B filler could be approximated by polynomial function third order, although certain deviations occur (Figure 6).

One of the characteristics of the synthesis route for epoxy-type composites is cross-linking of chemical bonds during the curing process. The reinforced particles are incorporated in the cross-linked net structure. Therefore, at an elevated temperature, chemical chains of final composite material start to be movable and the material becomes softer. In contrast to thermoplastic polymers, thermosetting polymers, like cured epoxies, do not melt at the higher temperature. In any case, one of the most important parameters for convenient application of composites with epoxy matrix is glass transition temperature (*Tg*). More precisely, this is the temperature region where mobility of the polymer chains increases significantly and the epoxy transitions from hard to soft material state occur.

**Figure 7:**
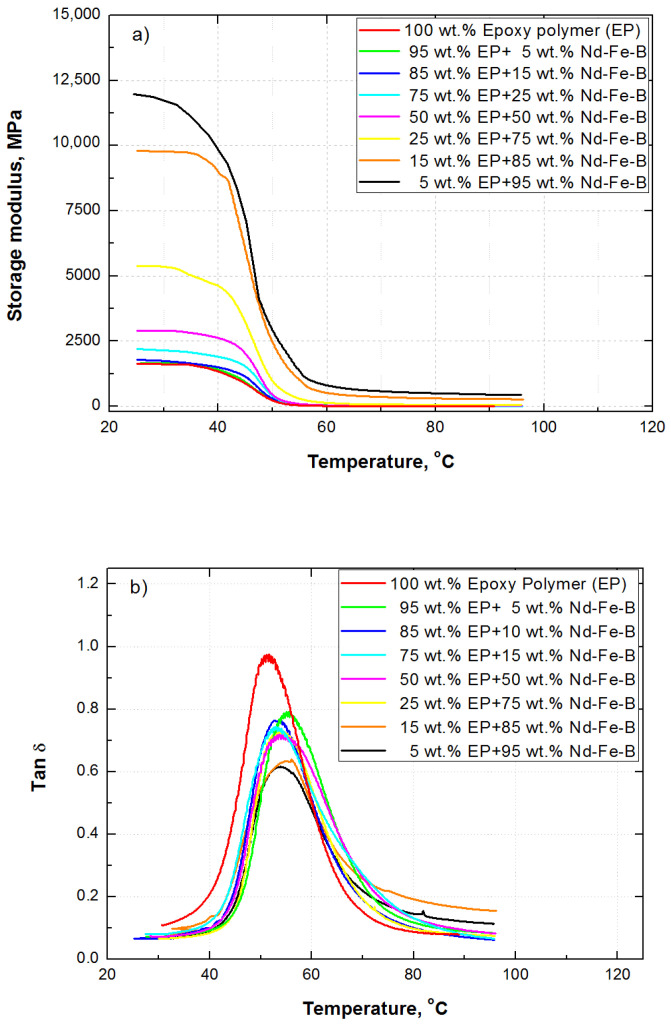
DMA curves of composites and pure epoxy resin, (**a**) Storage modulus and (**b**) Tanδ vs. temperature.

The glass transition of cured epoxy is difficult to measure by DSC, due to the confinement effect of the cured network on the movement of the polymer chain, although some transformations can be observed in the region around 50 °C in Figure 5. It is also more difficult for composites with lower epoxy content. Therefore, the samples were tested with DMA, which is a more sensitive technique than DSC. The material’s response to applied oscillating force on samples positioned at a three-point bending clamp was collected and analyzed. All the samples were tested at temperature intervals, from the glassy to the rubbery region. The results of the dynamic-mechanical measurements are presented in Figure 7 as a storage modulus (*E’*) and the Tanδ (*E”*/*E’*) (ratio of loss modulus (*E”*) and storage modulus) versus temperature for all synthesized composites and pure epoxy polymer.

The glass transition temperature is identified in the region between 47 °C and 52 °C as the midpoint of the “S” storage modulus curves (Figure 7a) and between 52 °C and 55 °C in the Tanδ curves (Figure 7b). The obtained values of *Tg* are closely connected with the cure temperatures. The epoxy system used is cured at the room temperature, which leads to the lower values of *Tg*. The higher *Tg* values can be achieved in epoxy systems by curing at the elevated temperature [46,47]. The *Tg* of the samples could also be moved towards higher temperatures using a different starting proportion of bisphenol component and hardener. An increase of 30 °C in the *Tg* could be achieved by doubling the ratio of components in the starting formulation, as reported by de Azúa et al. [47]. In the present work, such a step is not essential, due to fact that the target is not to achieve the highest performances of Nd-Fe-B/epoxy composites, but to make a comparison between composites synthesized at the same conditions, using the same particle size distribution filler in the epoxy matrix, by varying their content across a wide range.

The viscoelastic property of materials is described by the elastic component (storage modulus—*E’*), responsible for recovery after deformation, and the viscous component (loss modulus—*E”*), which represents the material’s ability to lose energy (as heat). The material’s loss tangent tanδ is the ratio of viscous to elastic components (*E”*/*E’*) and is sometimes referred to as the material’s damping ability [48].

A material with a higher storage modulus is stiffer and more resistant to deformation. As can be seen in Figure 7a, with a small addition of Nd-Fe-B particles, the storage modulus of the composites slightly increases. Composites with 50 wt.% of Nd-Fe-B show around 60% higher values of storage modulus compared to epoxy polymer. The composite with 75 wt.% of Nd-Fe-B has a significantly higher storage modulus, while drastic incensement is obvious for composites with 85 wt.% and 95 wt.% of Nd-Fe-B.

Overall, the dynamic modulus of composites in the glassy region strongly depends on nature, the physicochemical properties and concentrations of the epoxy polymer and Nd-Fe-B particles, and adhesion between them [49]. Above 70 °C, in the rubbery region, the hydrodynamic effect, the concentration and shape of the particles, their mutual interactions, and interactions with the epoxy matrix all have a direct impact on storage modulus, as reported elsewhere [9,50]. The values of the storage modulus are evidently very low in this region.

The results presented in Figure 7b show the tanδ of all tested samples is very low and practically constant at lower temperatures. With increasing temperature, the tanδ curves grow and pass through a maximum. The peak of pure epoxy polymer is sharper then the peak of composites, similar to the results of Atuanya et al. [51]. The highest loss tangent is shown by the pure epoxy resin (≈0.95) due to the largest presence of the polymer network, compared to the tested composites, which includes both internal friction and losses in the transition region (Figure 7b), as reported by Atuanya et al. [51]. With an increase in wt.% of Nd-Fe-B, the amount of epoxy network decreases, which results in a gradual decrease in the maximum of the tanδ in the transition zone to the glassy state (peak of the curves). Composite materials with 5, 15, 25, 50 I 75 wt.% of Nd-Fe-B have a tanδ of between 0.7 to 0.8. This is a higher viscous percentage than for composites with 85 and 95 wt.% of Nd-Fe-B filler (tanδ ≈ 0.6), which means that these samples will be able to absorb vibrations better and disperse them through the composite without failure.

### 3.3. Surface Properties

**Figure 8:**
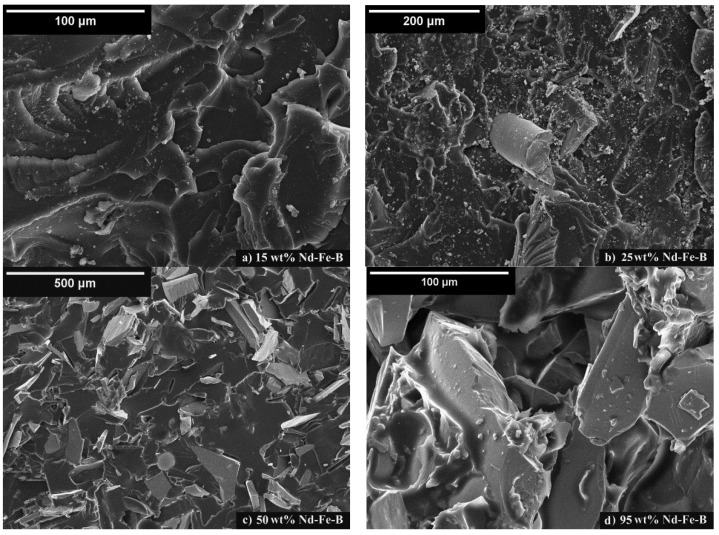
SEM images of the fracture surface of composites with (**a**) 15 wt.%, (**b**) 25 wt.%, (**c**) 50 wt.%, and (**d**) 95 wt.% of Nd-Fe-B magnetic particles in epoxy matrix.

SEM images of the fracture surface obtained via the impact testing process for composites with varying magnetic filler content are presented in Figure 8. Figure 8a,b show SEM images of the composites with 15 and 25 wt.% of magnetic filler in the epoxy matrix, respectively. A smooth fracture surface illustrates a relatively brittle behavior of the epoxy resin (indicated by the dark grey regions) with trapped angular Nd-Fe-B particles (indicated by the light gray regions). A minor concentration of particles induces isolation in the polymer matrix. A large isolation of particles causes each particle to act as a separate magnet.

Figure 8c shows the SEM image of the composite with a higher weight fraction of randomly distributed magnetic particles (50 wt.%) in the epoxy matrix. As illustrated in Figure 8c, a very clean surface exists, as a result of the impact test, in which some of the particles were drawn out (indicated by the black regions), while some of the particles were fractured.

This points out that a relatively strong adhesive force exists between the particles and the epoxy matrix. The overall energy resulting from the two opposing effects caused by particle pull-out and particle fracture led to optimum particle fraction (weight or volume) under maximum impact resistance of the epoxy-bonded Nd-Fe-B composites [52].

Figure 8d shows the SEM image of the composite with the highest observed weight fraction of Nd-Fe-B in the epoxy resin (indicated by the light gray colored region) and is identified at the surface of the particles. It can be noted that very fine particles have been incorporated into the epoxy resin. As a result, it was found that increasing the particle content certainly induces different surface structures. This results in different magnetic properties and impacts toughness which will be discussed in Section 3.4, as well as toughening (fatigue) mechanisms [33,53,54]. Since the smaller particles tend to fill in the pores between the large particles, this often results in increased magnetic composite density. Consequently, a smaller content of epoxy binder per composite volume can be attained [10]. A more detailed discussion related to particle shape effects on the packing density factor has been reported in previous work [17].

### 3.4. Magnetic and Mechanical Properties

**Figure 9:**
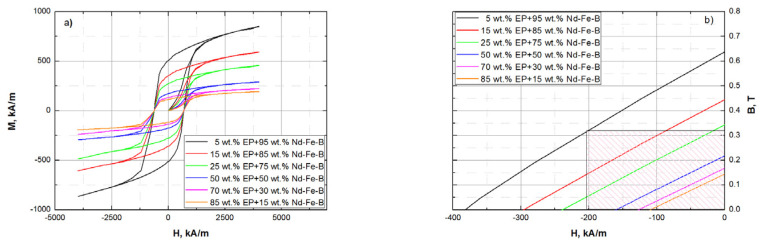
Hysteresis loops of magnetic composites in (**a**) M–H, and (**b**) B–H (second quadrant) diagram.

Figure 9 shows the magnetic behavior of all investigated Nd-Fe-B-epoxy-bonded samples when exposed to an external magnetic field. The Nd-Fe-B component is responsible for permanent magnetic properties presented as characteristic hysteresis loops in the magnetization-magnetic field strength (M-H) diagram (Figure 9a).

The largest hysteresis loop corresponds to the sample with the highest amount of Nd-Fe-B (95 wt.%) and, vice versa, the smallest hysteresis loop corresponds to the sample with the lowest amount of Nd-Fe-B (5 wt.%). With an increasing amount of Nd-Fe-B, hysteresis loops increase and, consequently, the magnetic properties of the samples increase. The magnetic measurements are conducted on an identical volume of samples, but weight ratios of Nd-Fe-B to epoxy were varied. Keeping this in mind, it is obvious that coercivity Hc shows identical values for all samples (Hc = 619.3 kA/m) because the coercivity of Nd-Fe-B is not dependent on weight or volume fraction [31,55]. On the other hand, the content of Nd-Fe-B has a direct impact on remnant magnetization and magnetization saturation, as presented in Figure 9a and Table 1.

It is known that a small volume of Nd-Fe-B has great magnetic energy, which is usually expressed as maximum energy product (BH)max [4]. To calculate (BH)max, a B-H diagram is constructed and presented in Figure 9b. (BH)max is calculated as the area of the highest rectangle under the B-H curve in the second quadrant [56]. This is illustrated in Figure 9b, for a sample with 95 wt.% of Nd-Fe-B and with the greatest magnetic properties, as a hatched pattern rectangle. The results summarized in Table 1 show that coercivity (Hcb), remanence (Br), and (BH)max increase with increasing content of Nd-Fe-B.

**Figure 10:**
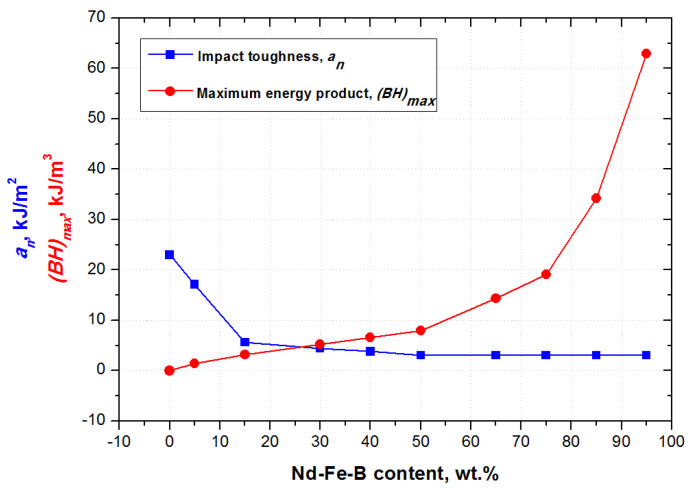
Impact toughness and maximum energy product versus Nd-Fe-B content.

The behavior of (BH)max and toughness, along with increasing wt.% of Nd-Fe-B particles, is presented in Figure 10. The linear enhancement of magnetic properties with an increased amount of Nd-Fe-B filler in the range from 10 wt.% to 70 wt.% is in agreement with the results of Kaidarova et al. [16]. However, our results when testing the highly filled PBMs (up to 95 wt.% Nd-Fe-B) also indicate that a slight increase of Nd-Fe-B filler concentrations beyond the 75 wt.% greatly impacts magnetic behavior. Highly filled composites above 75 wt.% show exponential growth of magnetic properties, as shown in Figure 10.

Values for impact toughness *a_n_* were taken directly from the impact tester and introduced as a function of the Nd-Fe-B weight fraction (Figure 10). The impact toughness was found to decrease from 9.3 to 1 kJ/m^2^, as the Nd-Fe-B magnetic particle content was increased from 10 to 95 wt.%. The pure epoxy resin also showed that the highest attainable impact toughness is 23 kJ/m^2^. Comparing the two curves presented in Figure 10, it is obvious that a contrasting trend is achieved, when varying Nd-Fe-B content, between impact toughness and maximum energy produced.

After analyzing the impact toughness results presented in Figure 10, the following three characteristics were found to be important for analyzing the influence of Nd-Fe-B weight fraction on fatigue crack growth parameters: (i) the 100 wt.% polymer (or the starting point), which correspond to the vertical part of the curve, (ii) the 15 wt.% of Nd-Fe-B, which corresponds to the inflection point, and (iii) the 50 wt.% of Nd-Fe-B, which corresponds to the horizontal linear part of the curve.

**Figure 11:**
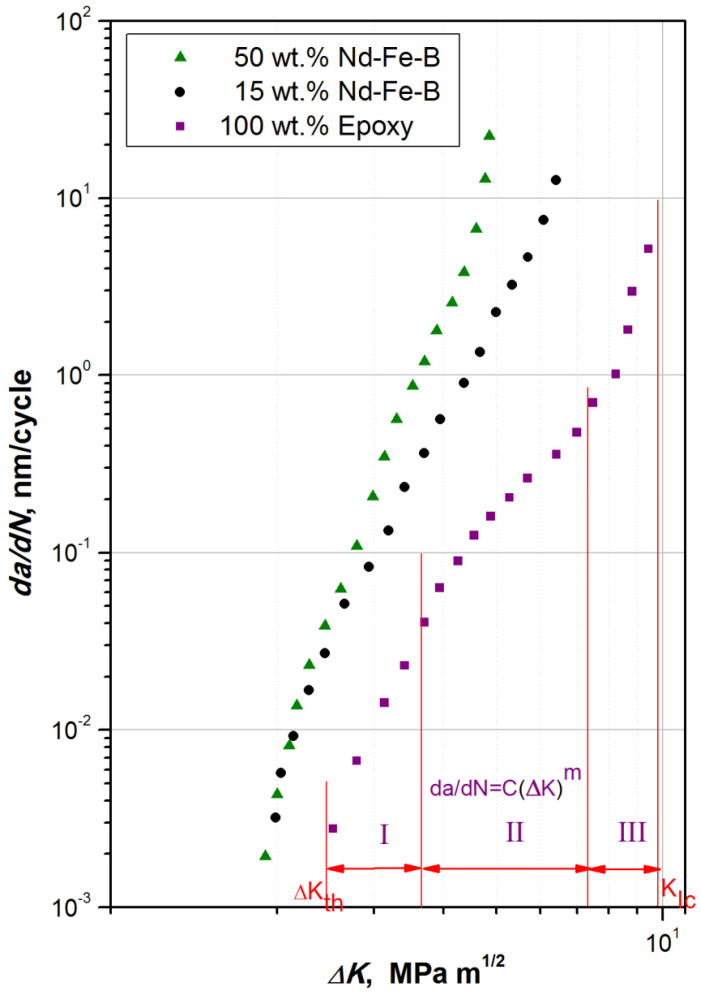
Diagram da/dN vs. Δ*K* for pure matrix and composites with 15 and 50 wt.% of Nd-Fe-B.

The experimental results obtained for the fatigue crack growth rate using these three different specimens are presented in Figure 11. The analysis associated with the three different curves can be presented as follows [33,54]:

1. In the short crack propagation area I, increasing Δ*K* causes a rapid increase in da/dN. Moreover, the threshold range of the stress intensity factor to fatigue threshold, Δ*K*_*th*_, is the point below which all fatigue cracks behave as cracks without the tendency to rise
2. The fatigue life, often referred to as “residual”, is a characteristic of the material, and can be determined from area II, as suggested by Paris and Erdogan [57]. Equation (1) can be used to express the linear relationship between Δ*K* and da/dN as follows [57]:
  (1) $$\frac{da}{dN}=C\cdot {\left(∆K\right)}^{m}$$
  where the constants *C* and m are empirically derived material properties that depend on the material, the stress range, and the test environment.
3. As for the fast crack propagation area III, there is a sudden crack growth before the final fracture. The critical value of fracture toughness *K_Ic_* for a given material is directly related to the early phases of a brittle fracture.

The values for *C* and m for a particular composite composition were obtained by constructing a straight trendline onto the log-log plot of the experimental data (in the linear area, also known as the “Paris” area, where m represents the slope and *C* represents the intercept). Table 2 shows that m value is the lowest for pure epoxy material (3.816). Moreover, m was found to increase with increasing filler contents. Increased m values are strongly connected with crack growth rate. Materials with low filler contents were found to have a lower fatigue crack growth rate and, consequently, lower values of m.

The value Δ*K* = 4 MPa m^1/2^ was determined from the stable crack growth region, in which the Paris law was found to be valid. Hence, this Δ*K* value was used to estimate the da/dN values for all three different specimen materials. The values of da/dN obtained were found to increase with increasing filler content, from 0.067 nm/cycle for the case of the pure epoxy specimen, to 0.6 nm/cycle for the case of the 85% epoxy specimen, all the way to 2.02 nm/cycle for the 50% epoxy specimen. It is obvious that, for the same Δ*K*, da/dN is shifted by one order of magnitude for materials with a higher content of filler.

The effect of Nd-Fe-B weight fraction (shown in Figure 11 and Table 2) also had great influence on crack propagation, with an obvious delay depicted for the pure epoxy sample (2.51 MPa·m^1/2^) compared to the composite cases containing 15 wt.% and 50 wt.% of Nd-Fe-B, respectively. The fatigue cracks were not able to propagate below 2.51 MPa·m^1/2^, which meant that materials under certain circumstances would not be affected, and can be fully exploited until the crack reaches its critical size of Δ*K_th_*.

## 4. Conclusions

This paper presents the effect of varying Nd-Fe-B weight fraction on maximum energy product, thermal properties and impact toughness of the final PBM product. Analyzing the thermal behavior of the samples, it is found that the degradation temperature is in the region from 305 °C to 365 °C. The highest ΔH of degradation temperature is indicated for the pure epoxy sample and subsequently decreases with a higher weight fraction of Nd-Fe-B filler, i.e., a smaller quantity of epoxy resin. According to Tanδ curves, the highest damping ability phenomena corresponds to the pure epoxy sample, while the lowest response is indicated for highly filled composites (85 and 95 wt.% Nd-Fe-B), as expected. The glass transition temperatures of the samples lie in the area between 52 °C and 55 °C, limiting their practical use. Storage modulus curves indicate the stiffness of material and show increasing values for highly filled composites, strongly influenced by microstructure properties, package density, particle–particle and particle–epoxy interactions. The magnetic properties follow this increasing trend. With increasing content of magnetic particles, magnetic properties increase, reaching a value of maximum energy product of 64.5 kJ/m^3^ for a composite with 95 wt.% Nd-Fe-B, which is almost twice the value of the composite with 85 wt.% Nd-Fe-B powder. SEM observations depicted that the plate-shaped particles are well incorporated into the epoxy matrix. Generally, the analysis of fracture surface indicates different structures for variously loaded composites, with a good particle to polymer adhesion and uniform particle distribution. In support of this, the results of FTIR spectroscopy do not indicate any chemical reaction between the magnetic filler and the epoxy matrix. Moreover, highly filled composite materials subjected to equal force levels resulted in lower impact toughness (up to 8 times lower) when compared to the pure epoxy case. Such results were found to be strongly connected to the fatigue crack propagation, the magnetic behavior, and the structural properties influenced by the parameters of processing. The determination of fatigue crack growth parameters was essential for the evaluation of materials’ fracture behavior, especially when a variable load was applied. The values of *K_Ic_* show that the fracture toughness decreases with higher Nd-Fe-B contents, since impact toughness also decreases.

## Figures and Tables

**Table 1:** Summary overview of the magnetic parameters of the composites.

Sample	Mr, kA/m	Ms, kA/m	Br, T	Hcb, kA/m	(BH)max, kJ/m^3^
5 wt.% EP + 95 wt.% Nd-Fe-B	511.2	854.7	0.64	381.8	64.5
15 wt.% EP + 85 wt.% Nd-Fe-B	358.2	591.2	0.44	295.1	33.9
25 wt.% EP + 75 wt.% Nd-Fe-B	271.8	452.4	0.34	237.3	20.9
50 wt.% EP + 50 wt.% Nd-Fe-B	178.2	292.4	0.22	159.3	8.73
70 wt.% EP + 30 wt.% Nd-Fe-B	136.5	226.5	0.17	125.7	5.41
85 wt.% EP + 15 wt.% Nd-Fe-B	112.4	191.8	0.14	108.6	3.91

**Table 2:** The parameters of fatigue crack growth.

Specimens	0 wt.% Nd-Fe-B 100 wt.% Epoxy	15 wt.% Nd-Fe-B 85 wt.% Epoxy	50 wt.% Nd-Fe-B 50 wt.% Epoxy
Parameters			
*C*	3.66·10^−4^	9.15·10^−5^	6.82·10^−5^
m	3.816	6.345	7.484
Δ*K_th_*, MPa·m^1/2^	2.51	1.99	1.85
Δ*K_Ic_*, MPa·m^1/2^	9.65	6.47	5.06
da/dN, nm/cycle (at Δ*K* = 4 MPa·m^1/2^)	0.067	0.6	2.02
Goodness of fit			
SSE	0.0001031	0.0003918	0.0061913
R-Square	0.9918	0.9974	0.9929

## Data Availability

Previously reported DSC data and SQUID magnetic measurements data for some composition of PBMs were used to support this study and are available at DOI:10.5772/18599 and DOI:10.2320/matertrans.M2011218. These prior studies (and datasets) are cited at relevant places within the text as references [9,17].
